# Morphological and molecular characterization of *Bitylenchus hispaniensis* (Nematoda: Telotylenchidae) from Iran

**DOI:** 10.21307/jofnem-2021-092

**Published:** 2021-11-03

**Authors:** Abbas Abdolkhani, Sedighe Azimi

**Affiliations:** 1 Department of Plant Protection, Faculty of Agriculture, Shahid Chamran University of Ahvaz, Ahvaz, Iran

**Keywords:** D2-D3-LSU, ITS rDNA, Khuzestan, Morphology, Morphometric data, Phylogeny, *Tylenchorhynchus*

## Abstract

During a survey on the biodiversity of plant-parasitic nematodes in Khuzestan province (southwest Iran), *Bitylenchus hispaniensis* was discovered around the rhizosphere of the euphrates poplar tree. The morphological and morphometric data were provided for the recovered species. To the best of our knowledge, this is the first report of *B. hispaniensis* from Iran and for the first time in association with euphrates poplar worldwide. Molecular phylogenetic analyses of the Iranian population of *B. hispaniensis* using the D2-D3 expansion segments of 28S rDNA and internal transcribed spacer (ITS rDNA) sequences using Bayesian inference (BI), showed a maximally supported clade with other sequences of the species.

The cosmopolitan genus *Tylenchorhynchus* (Cobb, 1913), is one of the biggest groups of plant-parasitic nematodes, which are migratory ectoparasites of the various plants (Siddiqi, 2000). The genus *Bitylenchus* (Filipjev, 1934) is very similar to the genus *Tylenchorhynchus*. The genus *Bitylenchus* is differentiated from *Tylenchorhynchus* in having areolated outer bands of lateral fields, a large postanal intestinal sac containing intestinal granules and fasciculi, relatively more thickened cuticle at the female tail tip, and gubernaculum lacking a crest (Gómez Barcina et al., 1992). These characters are reported in some species belonging to both *Tylenchorhyncus* and *Bitylenchus* (Handoo, 2000). The identification of species in *Bitylenchus* and *Tylenchorhynchus* remains a challenging task. Due to the certain species from one genus may come close to certain species from the other (Hosseinvand et al., 2020).

Some nematologists place *Bitylenchus* as a junior synonym of *Tylenchorhynchus* (Fortuner and Luc, 1987; Handoo, 2000; Geraert, 2011), but other nematologists recognized both as valid genera (Gómez Barcina et al., 1992; Siddiqi, 2000; Andrássy, 2007). In the study by Handoo et al. (2014) about integrative taxonomy of the genera *Bitylenchus* and *Tylenchorhynchus*, these two genera were clearly separated from each other and the monophyly of the genus *Bitylenchus* was accepted only after the exclusion of *B. ventrosignatus* (Tobar-Jiménez, 1969) Siddiqi, 1986. Hosseinvand et al. (2020), from their phylogenetic analyses in order to accept the hypothesis of *Bitylenchus* and *Tylenchorhynchus* as valid genera.

During a survey on nematodes of the Karkheh protected area in Khuzestan province, southwest Iran, *Bitylenchus hispaniensis* (Handoo, Palomares-Rius, Cantalapiedra-Navarrete, Liébanas, Subbotin & Castillo, 2014) was recovered. According to published literature, this is the first report of *B. hispaniensis* from Iran. The present study aims to characterize the Iranian population of *B. hispaniensis* based upon morphological and morphometric characteristics. Additionally, molecular data from LSU D2D3 and ITS rDNA markers were used to study the phylogenetic relationships with others *Bitylenchus* species.

## Materials and methods

### Nematode extraction and morphological observations

Several soil samples were collected from the rhizosphere of euphrates poplar (*Populus euphratica* Oliv.) trees in Khuzestan province, Iran. Centrifugal – flotation technique (Jenkins, 1964) or the tray method (Whitehead and Hemming, 1965) were used to extract the nematodes from soil samples. The collected specimens were killed in a hot 4% formaldehyde solution and transferred to anhydrous glycerin according to De Grisse (1969). Observations and measurements were conducted using a Leitz SM-LUX light microscope equipped with a drawing tube. Some of the specimens were photographed using an Olympus DP12 digital camera attached to an Olympus BX51 light microscope.

### DNA extraction, PCR and sequencing

For molecular analyses, single female specimens were picked out, examined in a drop of distilled water on a temporary slide under the light microscope, transferred to 3 μl of TE buffer (10 mM Tris-Cl, 0.5 mM EDTA; pH 9.0) on a clean slide, and then crushed using a cover slip. The suspension was collected by adding 20 μl TE buffer. The DNA samples were stored at –20°C until used as a PCR template. Primers for LSU rDNA D2-D3 ampliﬁcation were forward primer D2A (5′-ACAAGTACCGTGAGGGAAAGT-3′) and reverse primer D3B (5′-TCGGAAGGAACCAGCTACTA-3′) (Nunn, 1992). Primers for ampliﬁcation of ITS rDNA were forward primer rDNA1 (5′-TTGATTACGTCCCTGCCCTTT-3′) and reverse primer rDNA1.58S (5′-ACGAGCCGAGTGATCCACCG-3′) (Subbotin et al., 2000). The 30 μl PCR mixture contained 10 μl of distilled water, 15 μl of Master Mix (2X), 1 μl of each primer (10 pmol/μl), and 3 μl of DNA template. The thermal cycling program for amplification of both markers was as follows: denaturation at 95°C for 6 min, followed by 35 cycles of denaturation at 94°C for 30 s, annealing at 52.5°C (LSU D2-D3 primers)/54.7°C (ITS rDNA primers) for 30 s, and extension at 72°C for 60 s. A ﬁnal extension was performed at 72°C for 10 min. Amplification success was evaluated by electrophoresis on 1% agarose gel. The PCR products were purified using the QIAquick PCR purification kit (Qiagen^®^) following the manufacturer’s protocol and sequenced directly using the PCR primers with an ABI 3730XL sequencer (Bioneer Corporation, South Korea). The newly obtained sequences of the studied species were deposited into the GenBank database (accession numbers MZ725030/MZ725031 for LSU D2-D3 and MZ725020 for ITS rDNA).

### Phylogenetic analyses

The newly obtained sequences of the D2-D3 fragments of LSU rDNA and ITS rDNA and additional sequences of relevant species were selected after a BlastN search. The sequences were aligned by Clustal X version 2 using the default parameters (Larkin et al., 2007). The editing of both alignments was performed manually in MEGA7 program (Kumar et al., 2016). The base substitution model was selected using MrModeltest 2 (Nylander, 2004) based on the Akaike information criteria. A general time reversible model, including among-site rate heterogeneity and estimates of invariant sites (GTR + G + I), was selected for the both phylogenies.

The Bayesian analysis was performed to infer the phylogenetic trees using MrBayes v3.1.2 (Ronquist and Huelsenbeck, 2003), running the chains for four million generations. After to discard burn-in samples and to evaluate convergence, the remaining samples were retained for further analyses. The Markov chain Monte Carlo (MCMC) method within the Bayesian framework were used to determine equilibrium distribution and help estimate the posterior probabilities of the phylogenetic trees (Larget and Simon, 1999) using the 50% majority rule. Bayesian posterior probability (BPP) values higher than 0.50 are given on appropriate clades. The output ﬁles of the phylogenetic program was visualized using Dendroscope v3.2.8 (Huson and Scornavacca, 2012) and re-drawn in CorelDRAW software version 17.

## Results

### Systematics

#### Bitylenchus hispaniensis

**Figure 1:**
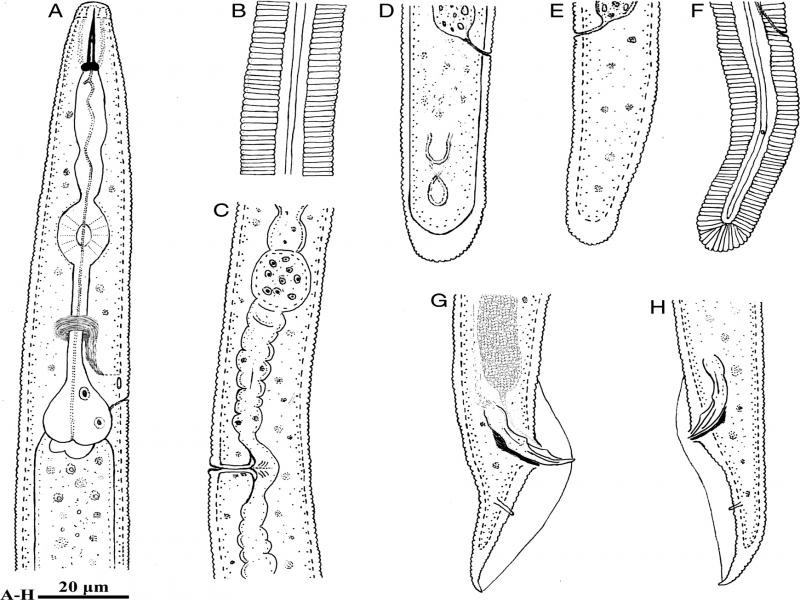
Line drawings of *Bitylenchus hispaniensis* from Iran. A-F: Female. A: Anterior body region; B: Lateral field at mid-body; C: Part of reproductive system; D-F: Posterior body region; G, H: Posterior body region of male.

**Figure 2:**
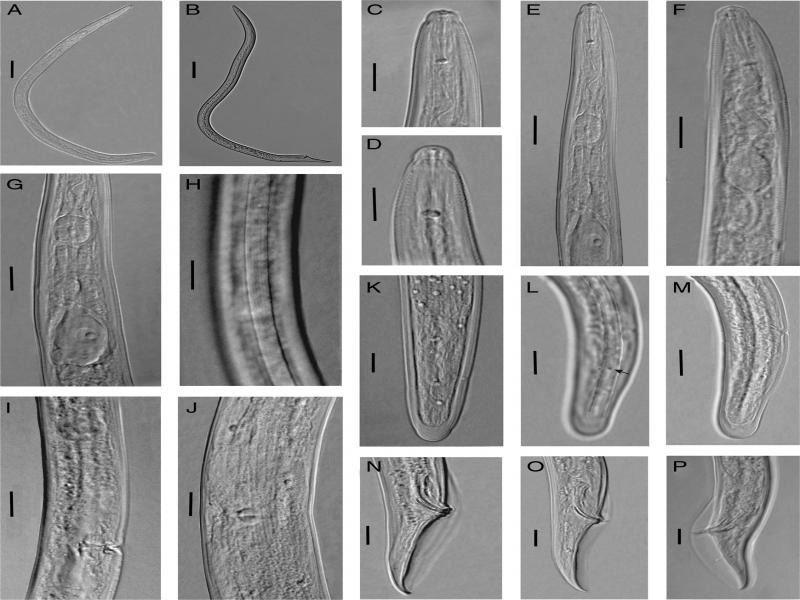
Light photomicrographs of *Bitylenchus hispaniensis* from Iran. A, C-M: Female. A: Entire body; C, D: Anterior body region; E-G: Pharyngeal region; H: Lateral field at mid-body; I, J: Vulval region; K-M: Posterior body region (the arrow indicates the phasmid); B, N-P: Male. B: Entire body; N-P: Posterior body region. (Scale bars: A, B = 50 μm; C-P = 10 μm).

(Figures 1–2; Table 1)

**Table 1.** Morphometrics of *Bitylenchus hispaniensis* from Khuzestan province, Iran and compare with the original description and Greek population.

	Khuzestan province, Iran		Handoo et al. (2014) Córdoba, Córdoba, Spain		Tzortzakakis et al. (2018) Crete, Greece
Character	Females	Males	Females	Males	Females
n	14	9	7	10	3
L	622.1 ± 41.0 (570–704)	661.7 ± 58.0 (591–763)	662 ± 38.4 (612–732)	640 ± 60.9 (548–760)	722 ± 47 (670–760)
a	25.6 ± 3.5 (21.8–2.8)	29.5 ± 0.1 (27.6–32.8)	30.4 ± 1.7 (27.8–32.5)	31.0 ± 3.9 (27.0–39.1)	32.3 ± 0.6 (31.9–33.0)
b	5.5 ± 0.4 (5.0–6.3)	5.9 ± 0.4 (5.3–6.5)	6.0 ± 0.4 (5.6–6.8)	6.1 ± 0.7 (5.4–7.8)	6.3 ± 0.4 (6.1–6.8)
c	13.3 ± 0.7 (12.8–15.7)	14.6 ± 0.7 (13.5–15.7)	14.2 ± 1.7 (11.8–16.8)	17.7 ± 1.4 (15.2–20.7)	14.9 ± 0.6 (14.4–15.5)
c′	2.8 ± 0.4 (2.5–3.2)	2.9 ± 0.2 (2.6–3.2)	2.9 ± 0.3 (2.4–3.3)	2.7 ± 0.2 (2.5–3.0)	3.0 ± 0.2 (2.7–3.2)
V	54.3 ± 1.5 (52.7–57.5)	-	54.6 ± 1.5 (52.5–57.0)	-	55.0 ± 1.0 (54–56)
Lip region height	3.7 ± 0.9 (3.2–4.1)	3.4 ± 0.3 (3.2–4.1)	3.6 ± 0.3 (3–4)	-	-
Lip region width	6.7 ± 0.2 (6.5–7.0)	6.4 ± 0.1 (6.0–6.5)	7.2 ± 0.3 (6.5–7.5)	-	-
Stylet length	17.5 ± 0.3 (17–18)	17.1 ± 0.4 (16.2–17.5)	16.1 ± 0.5 (15.5–17.0)	15.9 ± 0.8 (15.0–18.0)	17.3 ± 0.3 (17.0–17.5)
m	58.0 ± 2.7 (55.7–63.1)	54.2 ± 2.7 (48.2–57.3)	-	-	-
DGO	2.9 ± 0.2 (2.6–3.0)	2.8 ± 0.2 (2.5–3.0)	2.5	-	-
MB	49.2 ± 2.1 (46.8–52.6)	51.2 ± 4.0 (45.2–57.3)	-	-	-
Maximum body width	24.7 ± 7.4 (20.8–29.2)	20.8 ± 1.3 (18.8–23.4)	21.8 ± 1.0 (20.5–23.5)	20.7 ± 1.3 (18.0–23.0)	22.3 ± 1.2 (21–23)
Pharynx length	115.3 ± 6.7 (105–120)	116.0 ± 6.8 (106–124)	110.0 ± 6.5 (100–120)	105.4 ± 8.2 (90–120)	114.0 ± 5.3 (110–120)
Anterior end to median bulb	56.7 ± 3.8 (50.0–61.7)	56.9 ± 2.8 (52–58)	-	-	-
Anterior end to nerve ring	77.8 ± 2.9 (73.0–84.5)	80.0 ± 4.2 (75.0–87.7)	83.1 ± 5.6 (73–95)	-	-
Anterior end to excretory pore	93.3 ± 6.1 (85.5–104.0)	87.6 ± 9.0 (87–104)	89.7 ± 3.9 (85–95)	-	-
Body width at vulva	24.5 ± 2.7 (21.5–27.9)	-	-		-
Anal body width	16.5 ± 1.5 (15.0–19.5)	15.4 ± 1.5 (13.6–17.5)	16.1 ± 1.4 (13.5–17.5)	12.8 ± 0.8 (12–15)	16.5 ± 2.2 (14–18)
Tail length	47.4 ± 6.5 (37.0–55.2)	43.9 ± 3.5 (39.5–48.0)	47.4 ± 6.3 (37.5–57.0)	36.6 ± 5.6 (30–50)	48.3 ± 3.1 (45–51)
Tail annuli	51.8 ± 5.3 (40–58)	-	55.1 ± 8.1 (40–66)	-	-
Spicule length	-	25.5 ± 0.8 (24.0–26.5)	-	25.3 ± 1.8 (23–30)	-
Gubernaculum length	-	12.5 ± 0.4 (12.0–13.3)	-	11.4 ± 1.6 (10–15)	-

### Description

#### Female

Body arcuate ventrally to open C shape after heat fixation. Cuticle annuli 1–1.4 µm wide at mid-body. Lateral field with four incisures along the body, including the tail region, outer two incisures areolated. Lip region rounded, bearing 5–6 fine annuli, continuous to slightly offset from the body, cephalic framework slightly sclerotized. Stylet knobs rounded, laterally to posteriorly directed, 3–4  µm across. Median esophageal bulb elliptical to slightly oblong, 14.2 ± 1.2 (13.0–16.2)  µm long and 12.0 ± 1.1 (11.7–13.1)  µm wide, hemizonid usually two to three annuli anterior to excretory pore, almost 1.5 annuli wide, basal bulb pyriform, 25.0 ± 2.8 (20.0–29.2)  µm long and 14.8 ± 1.3 (14.0–16.5)  µm wide. Cardia well developed. Intestinal fasciculi present in the intestinal region. Reproductive system didelphic-amphidelphic, vagina 9–11  µm long, epiptygma absent, vulva a transverse slit, spermatheca rounded, filled with rounded sperm. Tail rounded hemispherical, tail terminus annulated, hyaline portion 6–8  µm. Phasmids located almost at the middle of the tail, at 22.4 ± 0.5 (21.5–23.1)  µm distance behind the anus. Post-anal intestinal sac absent.

#### Male

General morphology is similar to that of female except for character states associated with sexual differences. Tail conoid and pointed, enveloped by bursa. Spicules slightly curved ventrally. Gubernaculum well developed, half of the spicule length. The bursa is 61.5 ± 3.0 (52–65)  µm long.

#### Relationships

Among the *Bitylenchus* species that have been characterized molecularly, those that are close to *B. hispaniensis* include *B. dubius* (Bütschli, 1873) Filipjev, 1934, *B. huesingi* (Paetzold, 1958) Jairajpuri, 1982*, B. parvulus* (Hosseinvand, Eskandari, Ganjkhanloo, Ghaderi, Castillo & Palomares-Rius, 2020), *B. parvus* (Allen, 1955) Jairajpuri, 1982 and *B. serranus* (Gómez Barcina, Siddiqi & Castillo, 1992). Some characters separate *B. hispaniensis* from these species.

From *B. dubius,* by lip region continuous to slightly offset from the body bearing 5 to 7 annuli *vs* usually sharply offset from the body bearing 6 to 9 annuli and the absence of post-anal intestinal sac *vs* present. From *B. huesingi,* by tail bluntly rounded with a hemispherical to clavate terminus *vs* tail straight, cylindrical; and the absence of post-anal intestinal sac *vs* present. From *B. parvulus*, by cuticle anterior to vulva normal *vs* with irregular undulations (wrinkling) at its ventral side, intestinal fasciculi present *vs* absent, the absence of post-anal intestinal sac *vs* present, epiptygma absent *vs* present and tail bluntly rounded with a hemispherical to clavate terminus *vs* tail sub-cylindrical, abruptly narrowing near terminus giving a bluntly digitate appearance to its terminus. From *B. parvus*, it differs in the shape of the female tail being bluntly rounded with a hemispherical to clavate terminus bearing 40 to 66 annuli *vs* cylindrical tail with hemispherical terminus bearing 35 to 43 annuli; and in the absence of post-anal intestinal sac *vs* present. From *B. serranus*, by shorter stylet (15.5–18.0 *vs* 19–22  μm) and the absence of post-anal intestinal sac *vs* present.

#### Remarks

The general morphology of the recovered population of the species closely resembles the characters given for the type population (Handoo et al., 2014) and another population from Greece (Tzortzakakis et al., 2018). The type population from Spain and another population from Greece were extracted from the rhizosphere of olive. The presently studied species was recovered from the rhizosphere of euphrates poplar tree in the Karkheh protected area (GPS coordinates: 31°53ʹ49.308ʺN, 48°15ʹ33.3ʺE), Khuzestan province, southwest Iran. *B. hispaniensis* is herein reported for the first time in Iran and for the first time in association with euphrates poplar worldwide.

#### Molecular characterization and phylogenetic relationships

**Figure 3:**
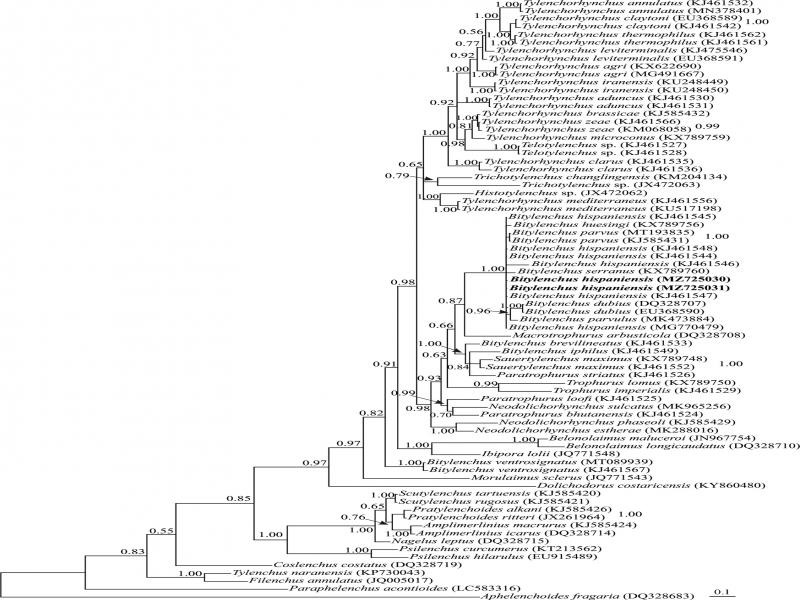
Bayesian 50% majority rule consensus tree inferred from analysis of the D2-D3 domains of the LSU rDNA sequences of Iranian population of *Bitylenchus hispaniensis* under the GTR + G + I model. Bayesian posterior probability values of more than 0.50 are given for appropriate clades. New sequences are indicated in bold.

Two 713 nt long D2-D3 expansion segments of LSU rDNA (MZ725030, MZ725031) were obtained for the Iranian population of this species. The BLAST search using these sequences revealed they have 99.85% identity with other sequences of the same species (MG770479, KJ461545 and KJ461547). A total of 74 sequences of Tylenchoidea Örley, 1880 and two sequences of Aphelenchoidea (Fuchs, 1937) Thorne, 1949 as outgroup taxa (LC583316 and DQ328683), were selected for the LSU phylogeny. This dataset comprised 796 total characters. The phylogenetic tree inferred using this dataset is presented in Figure 3. The newly generated sequences of the Iranian population of *B. hispaniensis* have formed a maximally supported clade with other sequences of the species in this tree.

**Figure 4:**
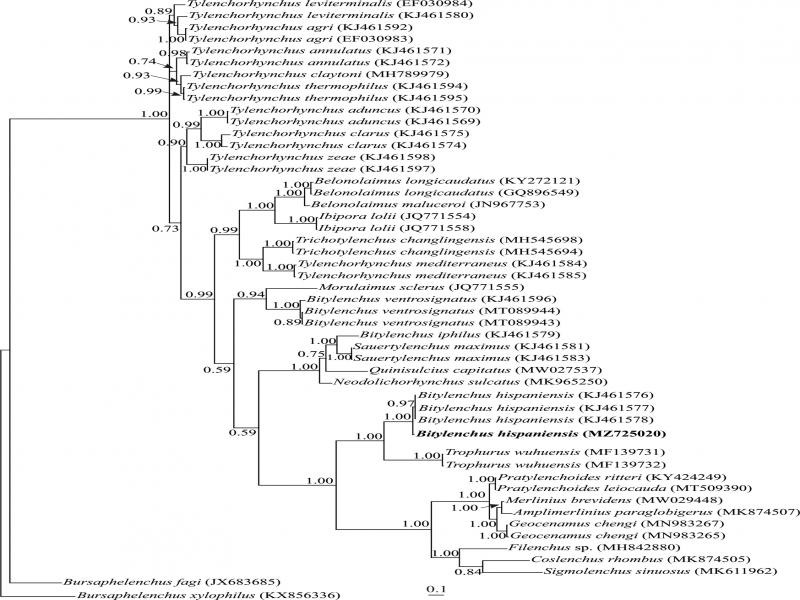
Bayesian 50% majority rule consensus tree inferred from analysis of the ITS rRNA gene of Iranian population of *Bitylenchus hispaniensis* under the GTR + G + I model. Bayesian posterior probability values of more than 0.50 are given for appropriate clades. New sequence is indicated in bold.

The amplification and sequencing of the ITS rDNA of the Iranian population of *B. hispaniensis* yielded one fragment with 532 nt long (MZ725020). The BLAST search using this sequence revealed it has 98.84% identity with another ITS sequence of the species (KJ461578). A total of 48 sequences of Tylenchoidea and two sequences of Aphelenchoidea as outgroup taxa (JX683685 and KX856336), were selected for ITS phylogeny. This dataset comprised 1140 total characters. The phylogenetic tree inferred using this dataset is presented in Figure 4. The sequence of the Iranian population of *B. hispaniensis* formed a maximally supported clade with other sequences of the species in this tree.

## Discussion

The objectives of this study were the morphological and molecular characterization of the Iranian population of *Bitylenchus hispaniensis* for the first time from Iran. The genus *Bitylenchus* is so similar to the genus *Tylenchorhynchus*. Due to the few and difficulties of morphological identifications in these two genera, the use of molecular markers for species identification is very important.

In the present study based on the 28S rRNA gene and ITS rRNA gene, *Bitylenchus* is paraphyletic. The results of the phylogenetic study by Handoo et al. (2014) indicated the monophyly for the genus *Tylenchorhynchus sensu* Siddiqi (2000), and monophyly for the genus *Bitylenchus sensu* Gómez Barcina et al. (1992) and Siddiqi (2000) was accepted after the exclusion of *B. ventrosignatus* from this genus. Hosseinvand et al. (2020), indicated that *Bitylenchus* species divide into two groups. Similar results were obtained by Shokoohi (2021). The molecular data of the other known species of *Bitylenchus* and *Tylenchorhynchus* will shed light on the phylogenetic relationships of their species and the closely related genera.
